# Plasma tau is increased in frontotemporal dementia

**DOI:** 10.1136/jnnp-2017-317260

**Published:** 2018-02-13

**Authors:** Martha S Foiani, Ione OC Woollacott, Carolin Heller, Martina Bocchetta, Amanda Heslegrave, Katrina M Dick, Lucy L Russell, Charles R Marshall, Simon Mead, Jonathan M Schott, Nick C Fox, Jason D Warren, Henrik Zetterberg, Jonathan D Rohrer

**Affiliations:** 1 Department of Molecular Neuroscience, UCL Institute of Neurology, London, UK; 2 UK Dementia Research Institute at UCL, London, UK; 3 Department of Neurodegenerative Disease, Dementia Research Centre, UCL Institute of Neurology, London, UK; 4 MRC Prion Unit at UCL, Institute of Prion Diseases, London, UK; 5 Clinical Neurochemistry Laboratory, Sahlgrenska University Hospital, Mölndal, Sweden; 6 Department of Psychiatry and Neurochemistry, Institute of Neuroscience and Physiology, The Sahlgrenska Academy at the University of Gothenburg, Mölndal, Sweden

## Abstract

**Background:**

Frontotemporal dementia (FTD) is a heterogeneous neurodegenerative disorder presenting clinically with personality change (behavioural variant FTD (bvFTD)) or language deficits (primary progressive aphasia (PPA)). About a third of FTD is familial with mutations in *GRN*, *MAPT* and *C9orf72* being the major genetic causes. Robust biomarkers of the underlying pathology are still lacking in FTD with no markers currently being able to distinguish those with tau and TDP-43 inclusions during life.

**Methods:**

This study used an ultrasensitive single molecule methodology to measure plasma tau concentrations in 176 participants: 71 with bvFTD, 83 with PPA and 22 healthy controls. The patient group included 36 with pathogenic mutations in either *MAPT* (n=12), *GRN* (n=9) or *C9orf72* (n=15). Group comparisons were performed between clinical and genetic groups and controls using a linear regression model with bias-corrected bootstrap CIs. Correlative analyses were performed to investigate associations with measures of disease severity and progression.

**Results:**

Higher plasma tau concentrations were seen in bvFTD (mean 1.96 (SD 1.07) pg/mL) and PPA (2.65 (2.15) pg/mL) compared with controls (1.67 (0.50) pg/mL). Investigating the PPA group further showed significantly higher levels compared with controls in each of the PPA subtypes (non-fluent, semantic and logopenic variants, as well as a fourth group not meeting criteria for one of the three main variants). In the genetic groups, only the *MAPT* group had significantly increased concentrations (2.62 (1.39) pg/mL) compared with controls. No significant correlations were seen with cross-sectional or longitudinal brain volumes, serum neurofilament light chain concentrations or disease duration.

**Conclusion:**

Plasma tau levels are increased in FTD in all clinical groups, but in the genetic subtypes only in *MAPT* mutations, the group of patients who definitively have tau pathology at postmortem. Future studies will be required in pathologically confirmed cohorts to investigate this association further, and whether plasma tau will be helpful in differentiating patients with FTD with tau from those with other pathologies.

## Introduction

Frontotemporal dementia (FTD) is a common cause of early onset dementia presenting clinically with behavioural change (behavioural variant FTD (bvFTD)) or language impairment (primary progressive aphasia (PPA)) and pathologically, with inclusions containing usually either tau or TAR DNA-binding protein 43 (TDP-43). However, clinicopathological correlation is poor and diagnosing the correct proteinopathy during life is difficult except when a genetic cause is present (mutations in *MAPT* cause tau pathology, while mutations in *GRN* and *C9orf72* cause TDP-43 pathology) or for certain phenotypes (eg, the semantic variant of PPA and FTD with motor neuron disease (MND) are both commonly associated with TDP-43 pathology).^1^

Current biomarkers (including cerebrospinal fluid (CSF) measures of tau and TDP-43) do not accurately identify the underlying proteinopathy in vivo in FTD, and therefore novel measures are being investigated,^1^ including assays utilising blood samples which are less invasive than CSF. Recently, an ultrasensitive methodology of measuring plasma tau has been developed which has shown raised concentrations in Alzheimer’s disease,^2^ but has yet to be examined in FTD.

In this study, we aimed to investigate whether the levels of plasma tau are increased in patients with FTD compared with healthy controls, and in particular whether they vary between FTD subgroups.

## Methods

### Study participants

Plasma was available from 176 consecutively recruited participants from the University College London FTD cohort studies: 71 participants with bvFTD^3^ (of which three had concurrent MND (FTD-MND)^4^), 83 with PPA^5^ (23 with the semantic variant of PPA (svPPA), 33 with the non-fluent variant of PPA (nfvPPA), 18 with the logopenic variant of PPA (lvPPA) and nine who did not fit diagnostic criteria for any of these subtypes, named PPA-not otherwise specified (PPA-NOS)^6^) and 22 healthy control participants (table 1).

**Table 1:** Demographics and plasma tau concentrations in patients and controls

	N	Age, mean (SD)	Gender, %male	Disease duration, mean (SD)	Tau (pg/mL), mean (SD)
bvFTD	71	64.2 (8.1)	79	6.2 (4.4)	1.96 (1.07)
PPA	83	67.1 (8.2)	59	4.9 (2.6)	2.65 (2.15)
*svPPA*	*23*	65.4 (7.2)	*52*	*5.5 (2.6)*	*2.47 (1.57)*
*nfvPPA*	*33*	69.7 (9.3)	*52*	*4.5 (2.7)*	*2.88 (2.88)*
*lvPPA*	*18*	66.1 (7.6)	*72*	*4.9 (2.5)*	*2.21 (1.22)*
*PPA-NOS*	*9*	64.1 (4.7)	*78*	*4.6 (2.0)*	*3.15 (1.76)*
Controls	22	68.7 (6.5)	41	N/A	1.67 (0.50)

PPA subgroups are shown in italics.

bvFTD, behavioural variant FTD; FTD, frontotemporal dementia; lvPPA, logopenic variant of PPA; non-fluent variant of PPA; PPA, primary progressive aphasia; PPA-NOS, PPA-not otherwise specified; svPPA, semantic variant of PPA.

Of the 155 participants with bvFTD or PPA, 37 (14 with bvFTD, seven with svPPA, 10 with nfvPPA and six with lvPPA) had also had CSF collected and analysed for β-amyloid (Aβ1–42), total tau (t-tau) and phosphorylated tau (p-tau181) using the INNOTEST assays (Fujirebio Europe, Gent, Belgium).

Age at sample collection and gender were significantly different in controls compared with bvFTD (younger and more men in bvFTD group) with no significant difference between controls and PPA (table 1). All further statistical analyses were therefore adjusted for age and gender. Disease duration at sample collection was not significantly different between groups (table 1). Thirty-six participants had a pathogenic mutation in the *MAPT* (12), *GRN* (9) or *C9orf72* (15) genes, and a subanalysis was performed comparing these genetic groups with controls. Mean (SD) age at sample collection was 58.6 (6.2) years for the *MAPT* group, 63.1 (3.3) for *GRN* and 65.1 (6.2) for *C9orf72*—both *MAPT* and *GRN* were significantly younger than controls. Ninety-two per cent of patients with *MAPT* mutations were male (significantly more than controls), while 56% of *GRN* and 73% of *C9orf72* cases were male (no significant difference from controls). No significant differences were seen in disease duration between the genetic subgroups: *MAPT* 6.8 (4.6) years, *GRN* 4.1 (3.3), and *C9orf72* 7.8 (4.3).

### Plasma tau measurements

EDTA plasma samples were collected from the participants, processed and stored at −80°C following standardised procedures. Plasma level concentrations were measured using the Human Total Tau kit (Quanterix, Boston, Massachusetts, USA) with the Simoa HD-1 Analyser (Quanterix, Boston, Massachusetts, USA). Briefly, samples, magnetic beads coated with Tau5 monoclonal capture antibody, and HT7 and BT2 monoclonal biotinylated detector antibodies were combined. Antibody epitopes in the mid-region of tau make the assay sensitive to normal and phosphorylated tau, including most of the known protein isoforms. Thereafter, the capture beads were resuspended with streptavidin-β-galactosidase (SBG) and resorufin β-D-galactopyranoside (RPG) and transferred to the Simoa disk. Each bead fits into a microwell in the disk and if tau has been captured the β-galactosidase hydrolyses the RGP substrate which generates a fluorescent signal whose concentration can be measured against a standard curve derived from known concentrations of recombinant tau. The lower limit of detection for the assay is 0.019 pg/mL and the intra-assay coefficient of variation was 4.7% (all samples were analysed in duplicate on one occasion using one batch of reagents).

### Correlative analyses

In order to assess the association of plasma tau with disease in FTD, a number of measures were also assessed. As measures of disease severity, disease duration was measured as the difference between the number of years between when the sample was taken and symptom onset, and cross-sectional brain volumes were assessed in those with available neuroimaging: of the patients with FTD, 111 (57 bvFTD, 20 svPPA, 29 nfvPPA and five PPA-NOS) had volumetric T1 MRI performed usually on the day of blood sampling (mean (SD) 0.0 (0.2) years from sample to scan), with cortical (frontal, temporal, parietal, occipital, insula and cingulate) and subcortical (hippocampus and amygdala) regions parcellated using an atlas propagation and label fusion strategy as previously described.^7^ As measures of disease progression, serum neurofilament light chain concentrations were available in 55 of the FTD patient cohort,^8^ and brain atrophy rate was measured in 32 patients (19 bvFTD, three svPPA, seven nfvPPA and three PPA-NOS) who had had follow-up brain imaging at a mean (SD) of 1.1 (0.2) years after the baseline scan.

Correlative analyses of plasma tau with CSF t-tau and p-tau181 were also performed.

### Statistical analysis

Plasma tau data were not normally distributed (D’Agostino-Pearson omnibus normality test); hence, groups were compared by examining contrasts between the group means using a linear regression model in STATA V.14 with 95% bias-corrected bootstrap CIs with 1000 replicates, adjusting for age and gender. The Spearman’s correlation coefficient was used to investigate the association between plasma tau in patients and each of the measures of disease severity and progression.

## Results

**Figure 1:**
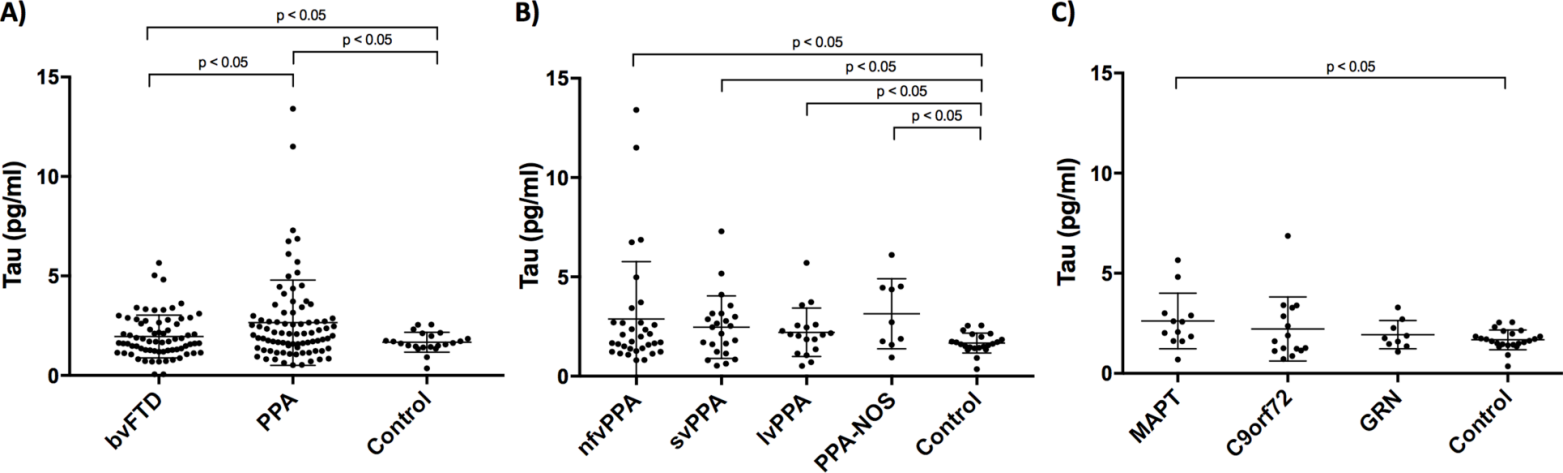
Plasma tau concentrations in participants comparing: (A) behavioural variant frontotemporal dementia (bvFTD), primary progressive aphasia (PPA) and healthy controls; (B) PPA subtypes: non-fluent variant (nfvPPA), semantic variant (svPPA), logopenic variant (lvPPA) and a group not fitting criteria for any of the three main variants, PPA-not otherwise specified (PPA-NOS) and (C) genetic subtypes: microtubule-associated protein tau (*MAPT*), *C9orf72* and progranulin (*GRN*).

The mean plasma tau concentration was significantly higher in both bvFTD (mean 1.96 (SD 1.07) pg/mL) and PPA (2.65 (2.15) pg/mL) compared with controls (1.67 (0.50) pg/mL) (figure 1a): adjusted difference in means for bvFTD versus controls (0.46; 95% CI 0.03 to 0.96), PPA versus controls (1.08; 95% CI 0.59 to 1.65). Concentrations were also significantly higher in PPA compared with bvFTD (0.62; 95% CI 0.15 to 1.30).

In a clinical subgroup analysis, concentrations in the PPA subgroups were 2.47 (1.57) pg/mL in svPPA, 2.88 (2.88) pg/mL in nfvPPA, 2.21 (1.22) pg/mL in lvPPA and 3.15 (1.76) pg/mL in PPA-NOS (figure 1b). All subgroups were significantly higher than controls: svPPA (0.80; 95% CI 0.38 to 1.55); nfvPPA (1.34; 95% CI 0.47 to 2.58); PPA-NOS (1.65; 95% CI 0.54 to 2.88); lvPPA (0.74; 95% CI 0.06 to 1.50). However, no significant differences were seen between disease groups.

In the genetic subgroup analysis, plasma tau concentrations were 2.62 (1.39) pg/mL in the *MAPT* group, 2.22 (1.60) pg/mL in the *C9orf72* group and 1.93 (0.70) pg/mL in the *GRN* group (figure 1c). Only the *MAPT* group had significantly higher concentrations than controls (adjusted difference in means 1.16 (95% CI 0.25 to 2.19); *GRN* vs controls 0.33 (95% CI −0.21 to 0.98); *C9orf72* vs controls 0.73 (95% CI −0.10 to 1.77)) with no significant difference between the disease groups.

ROC curve analyses showed a relatively poor ability to distinguish disease groups from controls for the majority of the clinical and genetic groups apart from PPA-NOS and *MAPT* which both had an area under the curve (AUC) of >0.75: AUC for bvFTD was 0.53, all PPA 0.65; svPPA 0.67, nfvPPA 0.60, lvPPA 0.65, PPA-NOS 0.77; *MAPT* 0.77, *GRN* 0.59 and *C9orf72* 0.51.

In the subgroup of 37 patients with CSF, nine had a t-tau/Aβ1-42 ratio of >1 (considered likely to be consistent with underlying Alzheimer’s disease pathology^9^: 0 bvFTD, one svPPA, two nfvPPA and six lvPPA) and the other 28 had a ratio of <1 (14 bvFTD, six svPPA, eight nfvPPA and 0 lvPPA). The mean (SD) plasma tau concentration was 2.31 (1.50) for the >1 group and 2.41 (2.45) for the <1 group. Both groups had tau levels significantly greater than controls (adjusted difference in means ratio >1 vs controls 0.84 (95% CI 0.05 to 2.12); ratio <1 vs controls 1.34 (95% CI 0.28 to 4.14)), but there was no difference between the two disease groups.

No significant correlations were seen between plasma tau concentrations and measures of disease severity (disease duration, r=0.19; cross-sectional brain volumes, r=0.05 to 0.19) or disease progression (serum neurofilament light chain, r=0.01; brain atrophy rate, r=−0.01 to 0.22).

No significant correlation was seen between plasma tau and either CSF t-tau (r=0.05) or p-tau181 (r=0.05).

## Discussion

Using an ultrasensitive detection method, we have demonstrated that concentrations of tau in the plasma are significantly increased in FTD. Clinically, both bvFTD and PPA (including each of the PPA subtypes) have higher concentrations than controls, but genetically, increased levels are only seen in the *MAPT* mutation group.

The only group in the study who definitively have tau pathology at postmortem are those with *MAPT* mutations so it is of particular interest that this group has raised levels compared with controls. Similarly, there were higher concentrations in those with nfvPPA who often have tau pathology (particularly those who develop an associated atypical parkinsonian syndrome as a few of the cohort here did). In contrast, patients with *GRN* and *C9orf72* mutations have TDP-43 pathology and do not have increased concentrations. However, svPPA is a TDP-43 proteinopathy in the majority of cases and this group also has significantly higher levels than controls. At present, the included groups do not have postmortem confirmation of the underlying pathology to further explore the ability of plasma tau to distinguish tau and TDP-43 proteinopathies, but such studies are needed to investigate this issue further.

Different *MAPT* mutations can have differential pathogenic effects on tau (eg, splice variants vs those that affect the structure and function of the protein). The current cohort of 12 cases contained eight with an intronic 10+16 mutation (the most common UK mutation), two with R406W and one each with P301S and Q351R mutations. Although too small numbers for a formal analysis, there were no clear differences between the 10+16 group (where levels varied from 0.70 to 5.66) and the other mutations. Further studies in larger cohorts of *MAPT* mutations will be required to understand whether there are any true differences in tau concentrations between different mutations.

Logopenic variant PPA is usually an atypical variant of Alzheimer’s disease. Raised plasma tau concentrations were seen in this group (and also in the subgroup for which the tau/Aβ1-42 ratio was >1) compared with controls, consistent with previous studies showing increases in typical Alzheimer’s disease.^2^ However, as with other studies, despite the significant group difference, there was extensive overlap between the patients and controls. In fact, this was the same for all analyses, with a large overlap in values between the disease groups and controls, likely precluding the use of plasma tau as a useful diagnostic biomarker.

This study did not investigate longitudinal changes in plasma tau, and it would be useful to explore this further to see how plasma tau changes with disease progression, including analysis in the very early stages of disease, as can be studied in presymptomatic genetic FTD cohorts such as the Genetic Frontotemporal dementia Initiative (GENFI).^7^ However, interestingly, in this cross-sectional study, plasma tau shows no significant correlation with either measures of disease severity or intensity, and therefore, at least in this heterogeneous cohort, does not seem to track with disease progression. It will be useful to investigate this further in more homogeneous and larger cohorts, for example, in those specifically with tau pathology. 7 The patient numbers in this study were too small to perform such subanalyses.

In summary, plasma tau concentration is increased in clinical subtypes of FTD but only those with *MAPT* mutations genetically. Further studies will be needed to investigate the longitudinal change in tau and whether, when applied to larger samples with pathological confirmation of tau burden, raised levels improve differentiation between patients with FTD with tau and TDP-43 pathology. However, while further studies may improve understanding of the pathophysiological relevance of plasma tau levels, the extensive overlap of levels with healthy controls is likely to limit its diagnostic utility.
